# Frontal ataxia: a frequent but underrecognized disorder in the spectrum of ataxias

**DOI:** 10.1055/s-0046-1820523

**Published:** 2026-05-12

**Authors:** Beatriz Cassarotti, Patrícia Aurea Andreucci Martins Bonilha, Thabata Emanuelle Martins Nunes, Léo Coutinho, Carlos Henrique Ferreira Camargo, Hélio Afonso Ghizoni Teive

**Affiliations:** 1 Universidade Federal do Paraná, Setor de Ciências da Saúde, Curso de Medicina, Departamento de Clínica Médica, Serviço de Neurologia, Curitiba PR, Brazil.; 2 Universidade Federal do Paraná, Setor de Ciências da Saúde, Curso de Medicina, Departamento de Clínica Médica, Programa de Pós-Graduação em Medicina Interna, Grupo de Doenças Neurodegenerativas, Curitiba PR, Brazil.

**Keywords:** Movement Disorders, Ataxia, Cerebellar Ataxia, Cerebral Small Vessel Diseases, Spinocerebellar Ataxias

## Abstract

**Background:**

Ataxia comprises a heterogeneous group of disorders with multiple clinical and etiological presentations. The frontal subtype, in particular, is poorly defined and often misdiagnosed, reflecting both its complex historical evolution and lack of formal diagnostic criteria.

**Objective:**

To evaluate clinical and epidemiological characteristics of a sample of patients with ataxia under follow-up in a private neurology clinic.

**Methods:**

We evaluated 48 patients diagnosed with ataxia over a 4-month period and followed them for 1-year in a private neurology clinic in southern Brazil. Clinical, neuroimaging, and laboratory data were analyzed. Patients were classified according to clinical and etiological subtypes based on criteria defined by the authors.

**Results:**

Frontal ataxia was the most frequent presentation (n = 15; 31.3%), predominantly affecting elderly patients with systemic arterial hypertension, diabetes mellitus, and MRI evidence of small vessel disease. These patients had a later disease onset (mean: 75.9 ± 8.8 years), lower SARA scores, and a nonprogressive course compared with degenerative cerebellar ataxias ( *p* < 0.0001). No correlation was observed between the severity of small vessel disease (Fazekas scale) and gait ataxia (rs = -0.28, *p* = 0.31). Hereditary ataxias, particularly spinocerebellar ataxias (SCAs), were the second most frequent group, followed by atypical Parkinsonian syndromes.

**Conclusion:**

Frontal ataxia emerged as a frequent and underrecognized subtype in routine neurological practice. These findings underscore the need for increased awareness and the development of evidence-based diagnostic criteria to better define and distinguish it within the spectrum of ataxic disorders.

## INTRODUCTION

The term *ataxia* is derived from the Greek word *taxis* , meaning *order* . Thus, *ataxia* describes a disorder characterized by impaired coordination and balance. Essential neural circuits that link the basal ganglia, cerebellum, and cerebral cortex are critical for coordinating movements of the eyes and limbs. 1 2

Cerebellar ataxia results from disruptions in afferent or efferent projections and manifests with gait ataxia, dysarthria, nystagmus, tremors, and cognitive dysfunction. 1 2 Beyond cerebellar pathology, disorders of the frontal cortex—particularly the frontal lobe—may also produce gait disturbances, classically referred to as frontal gait apraxia or frontal lobe ataxia. 3 4 5 6 7 This syndrome is characterized by a deliberate, wide-based, “magnetic” gait pattern and is often associated with conditions such as normal pressure hydrocephalus, vascular lesions, or neoplasms. 3 4 5 6 7 Frontal ataxia reflects both direct signs of frontal lobe dysfunction and disruptions of interconnected pathways, including the frontopontocerebellar (Arnold's) tract, basal ganglia circuits, the pedunculopontine nucleus (PPN), and the cerebellar dentate nucleus. 3 8 Similarly, disturbances in afferent sensory inputs can produce sensory ataxia, defined by limb or gait incoordination, impaired joint position and vibration sense, and Romberg's sign ( Table 1 ). 1 Together, these forms of ataxia illustrate the diverse mechanisms—cortical, cerebellar, and sensory—through which motor coordination can be compromised.

**Table 1:** Differential diagnosis of cerebellar, sensory, and frontal ataxias 2

Clinical features	Cerebellar ataxia	Sensory ataxia	Frontal ataxia
Gait ataxia	Present	Present	Present
Trunk ataxia/postural instability	Present	Present with advanced disease	May be present
Limb ataxia	Present	Lower limb dysmetria and/or dysdiadochokinesia	Absent
Bradykinesia	Absent	Absent	Present in the lower limbs
Dysarthria	Present	Absent	May be present
Frontal signs	Absent	Absent	Present
Nystagmus	Present	Absent	Absent
Ocular movement disorders	May be present	Absent	Absent
Reflexes	Normal or exacerbated	Hyporeflexia or areflexia	Normal
Vibratory sensibility	It may be reduced in advanced disease	Reduced/absent	Normal
Proprioception	Normal	Reduced/absent	Normal
Romberg sign	Absent	Present	Absent

The concept of frontal ataxia originated with Ludwig Bruns (1858–1916) in 1892, when describing balance and gait disturbances associated with frontal lobe lesions. 5 It was later expanded by Gerstmann, Schilder, and others to include features such as perseveration, incontinence, and gait apraxia. 4 5 Clinically, this syndrome is characterized by gait ataxia, retropulsion, and hypokinesia—predominantly affecting the lower limbs—with cognitive and frontal release signs, indicating both motor and executive dysfunction. Despite subsequent classifications, such as the Nutt–Marsden–Thompson framework, a universally accepted definition of frontal ataxia remains elusive, mirroring its complex historical evolution and heterogeneous clinical presentation. 2 3 4 5 6 7 Consequently, the diagnosis of frontal ataxia is often missed, misinterpreted, or entirely overlooked in many clinical series on ataxic disorders. 5

Assessing and diagnosing ataxias remains a significant clinical challenge, requiring not only a neurologist's expertise but also a thorough clinical history and detailed neurological examination. These steps are fundamental to guide the rational and cost-effective use of complementary tests, which becomes particularly relevant in clinical settings outside academic and teaching hospitals. In this context, the present study was designed to evaluate how patients presenting with ataxia were assessed and monitored over the course of 1 year in a private movement disorders clinic, thereby providing insights into diagnostic approaches and management strategies in routine practice, with particular attention to the accurate identification of frontal ataxia and its relative frequency compared with other subtypes.

## METHODS

### Selection of patients

We reviewed 578 medical records from patients treated at a movement disorders outpatient clinic in Curitiba, southern Brazil, from June to September 2022. Of that total, 417 (72.2%) presented with a variety of other movement disorders, 64 (11.1%) were diagnosed with ataxia, and 97 (16.7%) sought treatment for other neurological issues, primarily cognitive disorders.

**Fig. 1:**
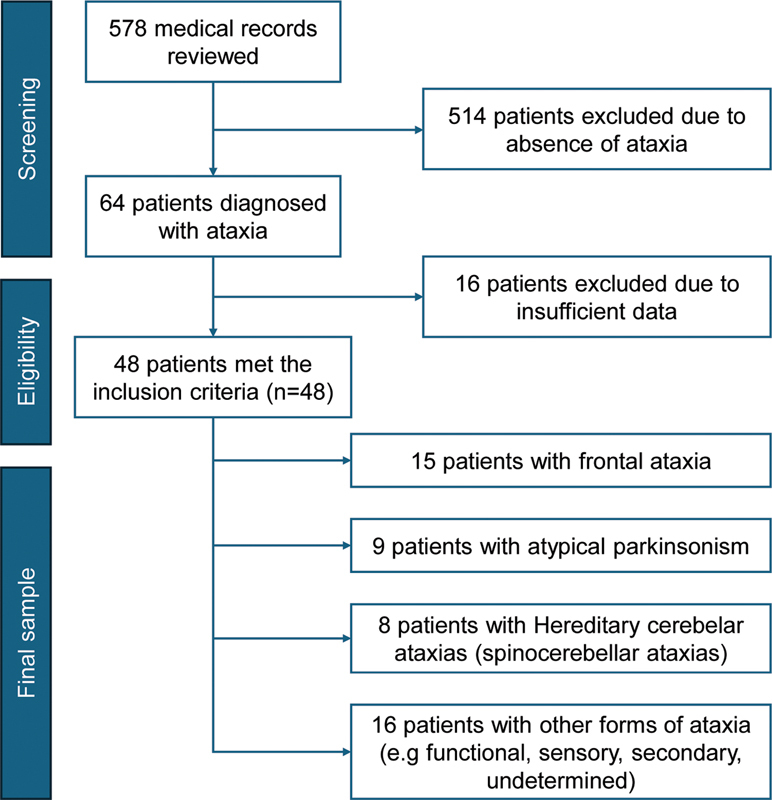
Note: Created in https://BioRender.com . Flow diagram illustrating patient selection and diagnostic classification.

A total of 48 patients were included in the study. Patients were eligible if they had a diagnosis of ataxia and met the following criteria: at least four consultations (one every 3 months) between September 2022 and September 2023, and both a confirmed syndromic diagnosis and a possible or confirmed etiological diagnosis of ataxia by the end of the study period. In cases where the etiology was uncertain due to the unavailability of genetic results or inconclusive findings from genetic, laboratory, or neuroimaging tests, patients were classified as having ataxia of undetermined cause. Records containing incomplete data were excluded from the analysis ( Figure 1 ).

### Ethical aspects

The project was approved by the Ethics Committee for Human Research of Hospital de Clínicas, Universidade Federal do Paraná (HC-UFPR), in accordance with the resolution of the HC-UFPR Ethics Committee for Human Research and Resolution no. 466/2012 of the Brazilian National Health Council (Conselho Nacional de Saúde, CNS, in Portuguese).

### Clinical evaluation and neuroimaging

A standardized questionnaire systematically gathered demographic data, clinical history, disease onset and progression, medication usage, additional examinations, comorbidities, and family history. The neurological examination was specifically tailored to assess this condition, utilizing the Scale for the Assessment and Rating of Ataxia (SARA). This scale evaluates eight domains to rate ataxia severity, yielding a score ranging from 0 (no ataxia) to 40 (most severe ataxia). The assessed factors include gait, stance, sitting posture, speech changes, dysmetria, the finger-to-nose test, rapid alternating hand movements, and heel-shin slide. 9 The ataxic clinical syndromes were defined according to Table 1 .

We employed the Montreal Cognitive Assessment (MoCA), a 30-point test evaluating cognitive and executive functions for cognitive screening. It includes six orientation questions, a five-word memory test, and a visuospatial assessment through clock drawing. Executive functions were evaluated using a simplified version of the Trail Making Test Part B, phonemic fluency, and a verbal abstraction task. An additional point was awarded to subjects with 12 or fewer years of formal education. 10

Patients underwent laboratory tests to supplement the diagnostic process, including complete blood count, basic biochemistry, thyroid-stimulating hormone (TSH) test, venereal disease research laboratory (VDRL) test, human immunodeficiency virus (HIV) test, ceruloplasmin, and vitamin levels. All participants were examined using a 1.5 T magnetic resonance imaging (MRI) brain scanner. Based on clinical indications and family history, molecular testing was performed for selected patients.

Frontal ataxia was defined as the presence of gait ataxia associated with executive dysfunction (inability to perform the MoCA Trail Making Test, cube copying, and a verbal fluency score <11), in combination with predominant frontal lesions of any etiology on brain MRI, late-onset, and no familial history. Ataxia occurring in the context of normal-pressure hydrocephalus (NPH) was also classified as frontal. Sensory ataxia was established in the presence of signs of impaired superficial or vibratory sensation in the lower limbs, or a Romberg sign, associated with nerve conduction studies demonstrating a sensory peripheral polyneuropathy. Ataxia was categorized as secondary when a definitive causal relationship with an external agent, systemic disease, or structural neurological lesion was established.

Diagnoses of autosomal dominant, recessive spinocerebellar, spastic, and myoclonic ataxias were considered based on clinical presentation and familial patterns and confirmed through positive molecular testing. Tandem repeat expansion (TRE)-specific tests were utilized for screening purposes, as a significant portion of hereditary ataxias is attributable to repeat expansions. Next-generation sequencing (NGS) techniques were employed in cases where TRE-specific tests were negative or for specific clinical scenarios. These included whole-genome (WGS), exome (WES), and targeted gene panel (TP) sequencing. 11 12

Established clinical criteria for conditions such as atypical Parkinsonism and functional ataxias were adhered to. 13 14 15

### Statistical analysis

The results were presented as means, medians, minimum and maximum values, and standard deviations (for quantitative variables) or as frequencies and percentages (for categorical variables). The normality of the variables was assessed using the Kolmogorov-Smirnov test. Analysis of variance (ANOVA) or nonparametric Kruskal-Wallis tests were used to compare the two or more groups regarding quantitative variables. Fisher's exact test was used to compare the groups regarding categorical variables. Spearman's correlation coefficient was used to evaluate the association between two quantitative variables. All *p-* values below 0.05 indicated statistical significance. The data were analyzed using the IBM SPSS Statistics for Windows (IBM Corp.) software, version 20.0.

## RESULTS

**Fig. 2:**
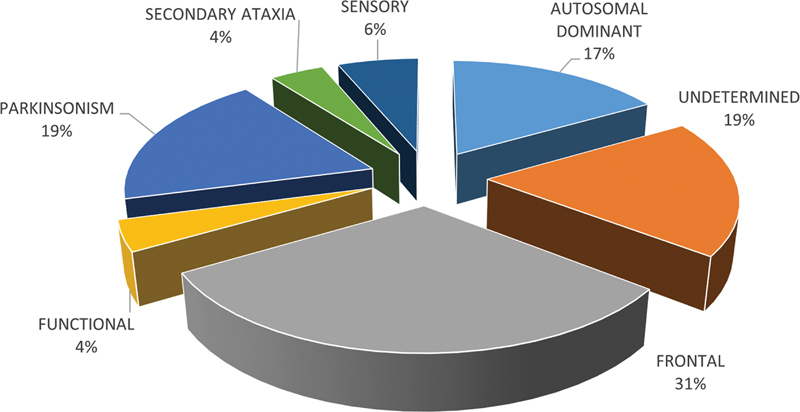
Notes: Autosomal Dominant: a group with spinocerebellar ataxias (SCAs) of autosomal dominant inheritance: 6 patients with SCA3, 1 with SCA6, and 1 with SCA10; Parkinsonism: a group consisting of 5 patients with multiple system atrophy and four patients with progressive supranuclear palsy. Distribution of patients evaluated according to etiology of ataxia (n = 48).

Among the 48 patients evaluated, 8 with autosomal dominant spinocerebellar ataxia (SCA) had their diagnoses confirmed through molecular testing: 6 had SCA3, also known as Machado-Joseph disease, 1 had SCA6, and 1 had SCA10. Atypical Parkinsonism was the second most common neurodegenerative cause of ataxia ( Figure 2 ). Multiple System Atrophy Cerebellar (MSA-C) was diagnosed in 5 patients, and progressive supranuclear palsy (PSP) in another 4.

Only one patient was diagnosed with secondary ataxia. He had epilepsy and showed slight improvement after discontinuation of chronic phenytoin therapy; cerebellar atrophy was evident on MRI. Sensory ataxia was diagnosed in three patients. Another two female patients, presenting with depression, anxiety, and an erratic pattern of ataxia, met DSM-5 criteria for conversion disorder and were diagnosed with functional ataxia ( Figure 2 ).

The most prevalent type of ataxia was frontal, which was found in 15 patients ( Figure 2 ). These patients, all older than 65 years, had a mean age of 79.3 ± 9.3 years and an average disease duration of 3.3 ± 1.3 years ( Table 2 ). They presented gait ataxia coupled with executive dysfunction and varying degrees of white matter vascular alterations in MRI. Despite these observations, no significant correlation was found between the severity of gait and cerebral vascular impairment, as measured by the Fazekas scale (rs = -0.28222, *p* = 0.30815). There was also no correlation between age (rs = 0.16744, *p* = 0.55086) or disease duration (rs = -0.4061, *p* = 0.1331) and SARA scores.

**Table 2:** Clinical and neuroimaging characteristics of patients with frontal ataxia

Patient - gender	Age (years)	Disease duration (years)	Clinical findings					Neuroimage findings		Assessment (SARA)	
			Gait ataxia	Trunk ataxia	Bradykinesia	Executive disfunction	Other cognitive dysfunctions	Fazekas	Ventricular enlargement	First	Follow-up
1 - M	85	5	+	+	+	+	−	2	−	5	5
2 - F	78	6	+	−	−	+	−	2	−	4	4
3 - M	74	4	+	−	−	+	+	2	−	6	6
4 - M	77	2	+	−	+	+	−	1	−	5	5
5 - M	84	4	+	−	−	+	+	3	−	4	4
6 - F	80	2	+	−	−	+	−	2	−	5	5
7 - M	86	2	+	−	−	+	−	2	−	6	6
8 - F	65	2	+	−	−	+	+	3	−	5	5
9 - M	89	4	+	−	−	+	+	2	−	5	5
10 - M	79	2	+	−	+	+	−	1	+	6	1
11 - F	81	4	+	+	−	+	−	2	−	4	4
12 - F	87	3	+	−	−	+	−	2	−	6	6
13 - F	84	4	+	+	+	+	+	2	−	4	4
14 - F	54	2	+	−	−	+	−	1	−	5	5
15 - F	86	4	+	+	−	+	+	2	−	6	6

Abbreviations: F, Female; M, Male; SARA, Scale for the Assessment and Rating of Ataxia; +, Present; -, Absent. Notes: Age and disease duration are presented in years. The time interval between the first evaluation and follow-up was one year. None of the patients in this group had clinical criteria for Parkinsonism.

None of the patients met the criteria for Parkinsonism, although four exhibited bradykinesia. The level of ataxia impairment remained stable in 14 patients. One patient with NPH had their condition resolved after surgery to implant a ventriculoperitoneal shunt ( Table 2 ). Excluding this NPH patient, all the remaining 14 (93.3%) patients with frontal ataxia had hypertension, 7 (46.6%) had diabetes, and 5 (33.3%) had dyslipidemia. The prevalence of hypertension and diabetes was significantly higher in this group compared to groups with other forms of ataxia ( Table 3 ).

**Table 3:** Comparison of the clinical characteristics of patients with the main types of ataxia

Variants		Frontal (n = 15)	SCA (n = 8)	Atypical Parkinsonism (n = 9)	*p* -value
Gender (female): n (%)(		8 (53.3%)	6 (66.6%)	5 (55.5%)	0.650 ^#^
Mean age (years)		79.3 ± 9.3	56 ± 11.6	75.7 ± 6.5	< 0.0001 ^*^
Mean age at onset (years)		75.9 ± 8.8	38.7 ± 6.0	70 ± 6.5	< 0.0001 ^*^
Disease duration (years)		3.3 ± 1.3	17.2 ± 8.2	5.6 ± 2	< 0.0001 ^**^
Mean SARA at first visit		5.06 ± 0.8	18.7 ± 4.6	22.5 ± 5.4	< 0.0001 ^***^
Mean SARA, after 1-year of follow-up		4.7 ± 1.3	20.8 ± 5.5	26.1 ± 5.3	< 0.0001 ^****^
Mean SARA disease progression		-0.33 ± 1.3	2.1 ± 1.5	3.5 ± 0.7	< 0.0001 ^*****^
Patient progression (SARA)		Improved 1 Worsened 0 Stable 14	Improved 0 Worsened 6 Stable 2	Improved 0 Worsened 9 Stable 0	< 0.0001 ^#^
Comorbidities: n	Diabetes	7	1	0	0.016 ^#^
	Hypertension	14	1	1	< 0.0001 ^#^
	Dyslipidemia	5	0	0	0.06 ^#^

Abbreviations: MSA, multiple system atrophy; PSP, progressive supranuclear palsy; SARA, Scale for the Assessment and Rating of Ataxia; SCA, spinocerebellar ataxia.

Notes: +Atypical Parkinsonism: a group consisting of 5 patients with MSA and four patients with PSP. SCAs of autosomal dominant inheritance: 6 patients with SCA3, 1 with SCA6, and 1 with SCA10. ^#^ Fisher's Exact Test; *ANOVA of the individual group comparison, only the comparison between frontal ataxia and ataxia associated with atypical Parkinsonism was not significant. **ANOVA of the comparison among groups, a statistical difference was observed only when comparing the group with SCAs to each of the others. ***ANOVA comparing patients with SCAs to those with ataxias with Parkinsonism, no statistical differences were observed when analyzed individually. ****ANOVA without statistical differences when the groups were compared individually. *****ANOVA without statistical differences when SCAs were compared with patients presenting with ataxias and Parkinsonisms.

Patients with frontal ataxia demonstrated the most favorable outcomes, showing the lowest SARA scores ( *p* < 0.0001) and overall clinical stability during the 1-year follow-up. Except for one patient with NPH who improved after surgery, no cases showed disease progression. In contrast, patients with atypical Parkinsonism and those with SCA presented higher SARA scores and a tendency toward worsening symptoms ( *p* < 0.0001). Although disease progression was faster in atypical Parkinsonism than in SCA ( *p* < 0.001), both groups exhibited comparable degrees of ataxia severity ( *p* = 0.156). Table 3 also shows that there were no significant differences in age at onset ( *p* = 0.38) or disease duration ( *p* = 0.38) between patients with frontal ataxia and those with atypical Parkinsonism. In contrast, individuals with SCA had the longest disease duration at the first assessment ( *p* < 0.001).

After extensive clinical investigations, nine patients had no identifiable etiology for their ataxia. One woman, with onset at 60-years-old and no family history, could initially be classified as idiopathic late-onset cerebellar ataxia (ILOCA). Another four patients showed an autosomal recessive inheritance pattern, and one had spastic ataxia in a sporadic form, but none obtained molecular confirmation. A TRE panel for recessive ataxias was requested for two of them—one was not performed, and the other yielded negative results. For another, WES was inconclusive, and WGS was later performed after WES identified a variant (6Kch37) in the HN66 gene; results were pending. A 91-year-old man presented with subacute, late-onset gait ataxia and vestibular syndrome; MRI showed only age-related changes. He received symptomatic treatment and physical therapy, with SARA improving from 7 to 0, but no definitive diagnosis was reached. Another patient had mild gait ataxia associated with Crohn's disease and mild cerebellar atrophy on MRI, but no causal relationship could be established. Finally, one patient with poorly controlled diabetes mellitus exhibited clinical features of sensory ataxia (hypopalesthesia and Romberg sign) despite normal electromyography findings.

## DISCUSSION

Most studies of patients with ataxia have been conducted in specialized research centers or neurogenetics laboratories, focusing on specific ataxia subtypes, particularly cerebellar ataxia, where referral bias often influences the clinical spectrum under investigation. 16 17 18 19 20 21 22 23 24 25 26 This study, therefore, provides a distinct perspective, highlighting the diagnostic profile of ataxias in routine clinical settings. Among the patients followed for 1-year, the most frequent etiology was frontal ataxia, particularly in older individuals with vascular risk factors and small vessel disease. These patients showed a shorter disease duration than those with SCAs and a more benign course compared with patients with neurodegenerative ataxias.

In this case series, 39 (81.3%) received a definitive diagnosis for their ataxia. The second most common subtype in this case series was SCAs, which reflects the higher prevalence of these diseases in Brazil, where SCA3 is the most common, as shown by numerous published studies. 18 21 23 26 This type of ataxia is also considered the most common worldwide. 16 19 22 24 25 Less frequently, patients in this cohort presented with sensory, secondary, or functional symptoms. Drug-induced ataxia was rare, with only one case attributed to chronic phenytoin use, likely reflecting both the declining prescription of phenytoin as an antiepileptic drug (AED) and the clinic's limited involvement in epilepsy and pediatric care. Other AEDs—including carbamazepine, oxcarbazepine, pregabalin, gabapentin, clobazam, ethosuximide, lacosamide, levetiracetam, topiramate, valproate, lamotrigine, and newer agents such as brivaracetam, cenobamate, perampanel, rufinamide, stiripentol, and eslicarbazepine—have also been associated with ataxia. However, many of these are not available in Brazil. Primidone and phenobarbital, frequently prescribed for tremors, are likewise known to cause ataxia as a common adverse effect; however, no such cases were observed in this sample. 27

In this study, frontal ataxia emerged as the most frequent subtype, identified in 15 patients (31.3%) who presented with late-onset gait disturbance, cognitive impairment, and vascular white matter changes, in the absence of clinical or neuroimaging findings suggestive of neurodegenerative disease or a family history of ataxia. The diagnosis was based on clinical criteria defined by the authors, emphasizing the combination of gait ataxia, executive dysfunction, and MRI evidence of small vessel disease. 2 3 6 7 These patients, predominantly elderly (mean age: 79 years), had a relatively short disease duration (mean 3 years) and mild SARA scores, remaining clinically stable throughout follow-up. Unlike patients with degenerative ataxias, they showed no evidence of progression. Vascular risk factors were common—particularly systemic arterial hypertension and diabetes mellitus, both statistically significant—and neuroimaging typically revealed small vessel disease with Fazekas scores between 2 and 3. Mild bradykinesia was occasionally observed, but without the presence of Parkinsonism. These findings support the notion that in our population, frontal ataxia represents a vascular-related gait disorder within the spectrum of noncerebellar ataxias. 6

However, this interpretation has been questioned. The presence of white matter lesions on MRI does not necessarily indicate a vascular etiology. For example, pseudoatrophy and ependymal transudation in NPH can mimic the clinical presentation of frontal ataxia—gait instability, bradykinesia, and cognitive dysfunction—but require distinct therapeutic approaches. Misclassifying such patients as having vascular ataxia could lead to inappropriate management. Moreover, history of vascular risk factors alone is not decisive and, in many of these cases, the distribution of white matter lesions is diffuse rather than predominantly frontal. Several studies have also failed to demonstrate a consistent association between Parkinsonism, gait disturbance, and cerebral small vessel disease. 28 The lack of correlation between gait severity, as measured by the SARA score, and the extent of vascular lesions on MRI in our series further supports this view. In our series, only one patient was diagnosed with NPH and showed marked improvement after ventriculoperitoneal shunting, following established diagnostic and treatment guidelines. 29 In contrast, for presumed vascular cases, no MDS-endorsed clinical definition currently exists, making consistent classification and epidemiological assessment difficult. 3 4 5 6 7

In clinical practice, patients with frontal ataxia are often misdiagnosed as having vestibular disorders, ILOCA, Parkinson's disease, or atypical Parkinsonism. The broad-based, “magnetic” gait typical of frontal ataxia frequently contributes to diagnostic confusion. 2 4 However, the presence of frontal release signs—together with the absence of cerebellar dysarthria, nystagmus, Parkinsonian rigidity, resting tremor, or bradykinesia affecting the upper limbs and face—constitutes a distinctive semiological profile suggesting a frontal rather than cerebellar or basal ganglia origin. 4 These patients differ from those with progressive, adult-onset, nonfamilial ataxia, who may have acquired or genetic etiologies but often remain undiagnosed and are ultimately classified as ILOCA. Among such cases, MSA-C accounts for approximately 30 to 40% and represents the most frequent neurodegenerative cause. 17 In our series, the remaining patients with ataxia were diagnosed with atypical Parkinsonian syndromes, including MSA-C and PSP. Notably, however, unlike these neurodegenerative conditions, frontal ataxia in our cohort was nonprogressive, had a later onset, and exhibited milder severity scores, reinforcing its distinct clinical profile.

In our cohort, all patients presented frontal ataxia secondary to vascular disease. However, considering Bruns' pioneer description of an ataxic patient with a frontal lobe tumor, we understand that any frontal lobe pathology might cause ataxia, such as demyelinating diseases, neoplasms, central nervous system (CNS) infectious disorders, and leukodystrophies.

Finally, we should discuss the use of the terms apraxia and ataxia, which are commonly used to describe gait abnormalities in these patients. From a historical perspective, these terms were often used interchangeably by various authors, leading to significant confusion. While apraxia is a disorder of motor planning, with magnetic, broad-based gait, ataxia is a disorder of balance, leading to postural instability. 3 4 7 As described by Bruns and other classical authors, these features might coexist in patients with frontal lobe pathology. 5 6 In our study, the term “Frontal ataxia” was used as an umbrella term to encompass all gait abnormalities, since ataxic gait dominated the clinical picture.

This study has several limitations. First, data were collected from a private neurology clinic with a relatively small sample size, which introduces selection bias and limits the external validity of the findings. The relatively short follow-up may also have led to an overestimation of the benign evolution observed in patients with frontal ataxia relative to other forms. Replication in larger and more diverse populations to better define the epidemiology of ataxias in everyday clinical practice. Another important limitation is the lack of molecular genetic testing for all patients, particularly for elderly individuals with frontal ataxia and those with a possible diagnosis of autosomal recessive cerebellar (ARCA) type. Nevertheless, hereditary late-onset cases are rare in this age group, which partially mitigates this limitation. Cognitive screening tests were also used to infer executive dysfunction, although a more comprehensive neuropsychological evaluation was not conducted. Moreover, the clinical criteria applied to define frontal ataxia were developed by the authors themselves, reflecting the absence of validated diagnostic standards for this syndrome. As a result, direct comparisons with other published series were not possible.

In conclusion, frontal ataxia emerged as the most frequent subtype in a clinical setting outside specialized research centers, followed by neurodegenerative causes such as SCAs and atypical Parkinsonian syndromes. This finding challenges the traditional view that hereditary or degenerative ataxias predominate. It raises the question of how often frontal ataxia—still lacking formal diagnostic criteria—is overlooked or misdiagnosed in routine neurological practice. Patients identified with this syndrome were older, had vascular risk factors and small vessel disease, and exhibited a milder, nonprogressive course compared with degenerative cases. These results underscore the clinical relevance of frontal ataxia and highlight the need for greater recognition of its distinctive features. Whether it represents a distinct clinical entity or an underdiagnosed manifestation of other disorders remains uncertain; however, this study calls for further large-scale investigations to establish evidence-based diagnostic criteria.
